# Capacity of the health facilities to manage Alzheimer’s and related dementia diseases in Mukono district: Challenges and recommendations

**DOI:** 10.1192/j.eurpsy.2023.412

**Published:** 2023-07-19

**Authors:** D. Jephthah, I. Ddumba

**Affiliations:** ^1^Nursing, Victoria University; ^2^Research and Innovation, African Research Center 4 Ageing & Dementia, Kampala, Uganda

## Abstract

**Introduction:**

With the projected increase in the number of older persons in both low and middle-income countries, the burden of Alzheimer’s and related dementia diseases (AD/ARDs) is projected to increase as well. However, the health systems are inadequately prepared to offer optimal care for patients with AD/ARDs, despite the growing disease burden.

**Objectives:**

The aim of this study was to assess the capacity of the health facilities to optimally manage Alzheimer’s and related dementia diseases in the Mukono district

**Methods:**

We conducted a cross-sectional between August and December 2018. A survey of 32 facilities (3 hospitals, 2 health center IV (HCIV), 15 health center III (HCIII) and 6 health center II (HCII), and 6 Private health facilities) in Mukono district. We conducted a thorough assessment of medical records, interviewed heads of the facilities, and a questionnaire was administered to 46 health workers. The study assessed the service provision for AD/ARDs, Knowledge of AD/ARDs management, challenges, and opportunities.

**Results:**

Out of 32 health facilities assessed, 4 in 10 (42%) facilities reported managing (diagnosing/treating) clients with AD/ARDs, and the majority (90.2%) were run by Non-Physician Health Workers (NPHW). Only 2 in 10 had guidelines for managing AD/ARDs. Less than half (46.4%) had AD/ARDs medicines in stock (mainly Haloperidol) and all of the private facilities lacked essential medicine to treat AD/ARDs. All health center IIs lacked drugs for AD/ARDs. A significant knowledge gap in assessing and diagnosing AD/ARDs was observed among all the health workers. All health workers highlighted the need for additional training in AD/ARDs. A multitude of client and health provider challenges were observed in this study

**Conclusions:**

Health facilities in Mukono district are inadequately prepared to offer optimal services for the management of AD/ARDs. AD/ARDs drugs, knowledge gap, and human resources for health presented a great challenge. In order to address the inadequately capacity to manage AD/ARDs, emphasis should be dwelt on strengthening the health facilities.

**Disclosure of Interest:**

None Declared

